# Case Report: A unique case of Philadelphia chromosome-positive mixed-phenotype acute leukemia with active Epstein-Barr virus infection in a kidney transplantation recipient

**DOI:** 10.3389/fonc.2026.1865977

**Published:** 2026-07-14

**Authors:** Mengping Chen, Xuelian Hu, Weiying Feng, De Zhou

**Affiliations:** 1 Department of Hematology, Shaoxing People’s Hospital, Shaoxing, China; 2 Department of Hematology, the First Affiliated Hospital, Shaoxing University, Shaoxing, China; 3 Department of Hematology, the First Affiliated Hospital, Zhejiang University School of Medicine, Hangzhou, China

**Keywords:** Epstein Barr virus infection, kidney transplantation, leukemia, mixed phenotype, Philadelphia chromosome

## Abstract

Philadelphia chromosome-positive (Ph+) mixed-phenotype acute leukemia (MPAL) is a rare entity of acute leukemia. The co-occurrence of Ph+ MPAL and active Epstein–Barr virus (EBV) viremia in a renal transplantation recipient represents an exceptionally rare and clinically challenging scenario. Here, we report a case of Ph+ MPAL arising concurrently with EBV infection in a patient who had undergone kidney transplantation nine years earlier. A tailored therapeutic strategy was implemented, combining olverembatinib with dexamethasone, lisaftoclax and azacitidine for leukemia treatment, alongside B-cell–depleting therapy with rituximab for EBV viremia. This integrated approach successfully induced hematologic remission and achieved clearance of EBV viremia, providing a potential therapeutic framework for managing this complex dual condition. To our knowledge, this is the first reported case of this specific clinical triad, highlighting the significant therapeutic challenges posed by the coexistence of these conditions.

## Introduction

Mixed-phenotype acute leukemia (MPAL) is a rare and highly heterogeneous subtype of acute leukemia. It is defined by either the co-expression of myeloid and lymphoid lineage markers within a single blast population (biphenotypic) or the presence of distinct blast populations of different lineages (bilineal) (1). Among its subtypes, B/myeloid MPAL is the most common, followed by T/myeloid MPAL, while B/T or trilineage phenotypes are exceedingly rare (2). The Philadelphia chromosome, resulting from the t(9,22)(q34;q11.2) translocation and generating the BCR-ABL fusion oncogene, is a well-established driver in chronic myeloid leukemia (CML) and B-cell acute lymphoblastic leukemia (B-ALL), but is infrequently observed in MPAL, where it defines a distinct and aggressive subset (3). Previous studies have reported that MPAL accounts for approximately 2%–3% of all acute leukemias, whereas Philadelphia chromosome-positive (Ph+) MPAL represents an even rarer subset, comprising less than 1.0% of cases (2, 3). Due to its rarity and biological heterogeneity, the pathogenesis, morphologic features, and molecular characteristics of MPAL remain incompletely understood, and standardized treatment strategies have yet to be established. MPAL is considered a high-risk form of leukemia with increased relapse rates and poor long-term survival (4).

Epstein-Barr virus (EBV), a member of the human herpes virus family, is a double-stranded DNA virus with a strong tropism for B lymphocytes (5). It can infect over 90% of the global population. The host immune response, including both cellular and humoral effectors, plays a central role in limiting the primary infection and controlling the lifelong viral carrier state in which latently infected B cells constitute the reservoir (6–8). In organ transplant recipients, primary or secondary EBV infection (reactivation/reinfection) may occur first via natural routes of transmission, second through transmission from the donated organ or blood transfusion, or third as a consequence of intensive immunosuppression. Chronic immunosuppression is a well-recognized risk factor for EBV reactivation and the subsequent development of post-transplant lymphoproliferative disorders (PTLD), a spectrum of EBV-driven lymphoid proliferations (9). However, the co-occurrence of EBV infection and *de novo* acute leukemia, distinct from the classical PTLD, after transplantation is extremely rare, and such case presents significant challenges for clinical management. These challenges underscore the need for optimized management strategies and individualized therapeutic approaches in this complex setting.

## Case presentation

A 50-year-old female was admitted to the hospital with abnormal blood counts on August 6, 2025. Routine labs revealed significant leukocytosis, anemia, and thrombocytopenia. Over the preceding three months, the patient exhibited a mild elevation in white blood cell count. On admission, she had no fever, dizziness, or weakness. On physical examination, she appeared pale, without evidence of hepatosplenomegaly. She had no family history of hematologic malignancies or renal diseases. She had a history of end-stage renal disease and underwent a kidney transplantation nine years earlier. Her maintenance immunosuppressive regimen included tacrolimus, mycophenolate mofetil, and prednisone. She also had a more than 9-year history of chronic hepatitis B infection and was receiving long-term antiviral therapy with entecavir.

**Figure 1:**
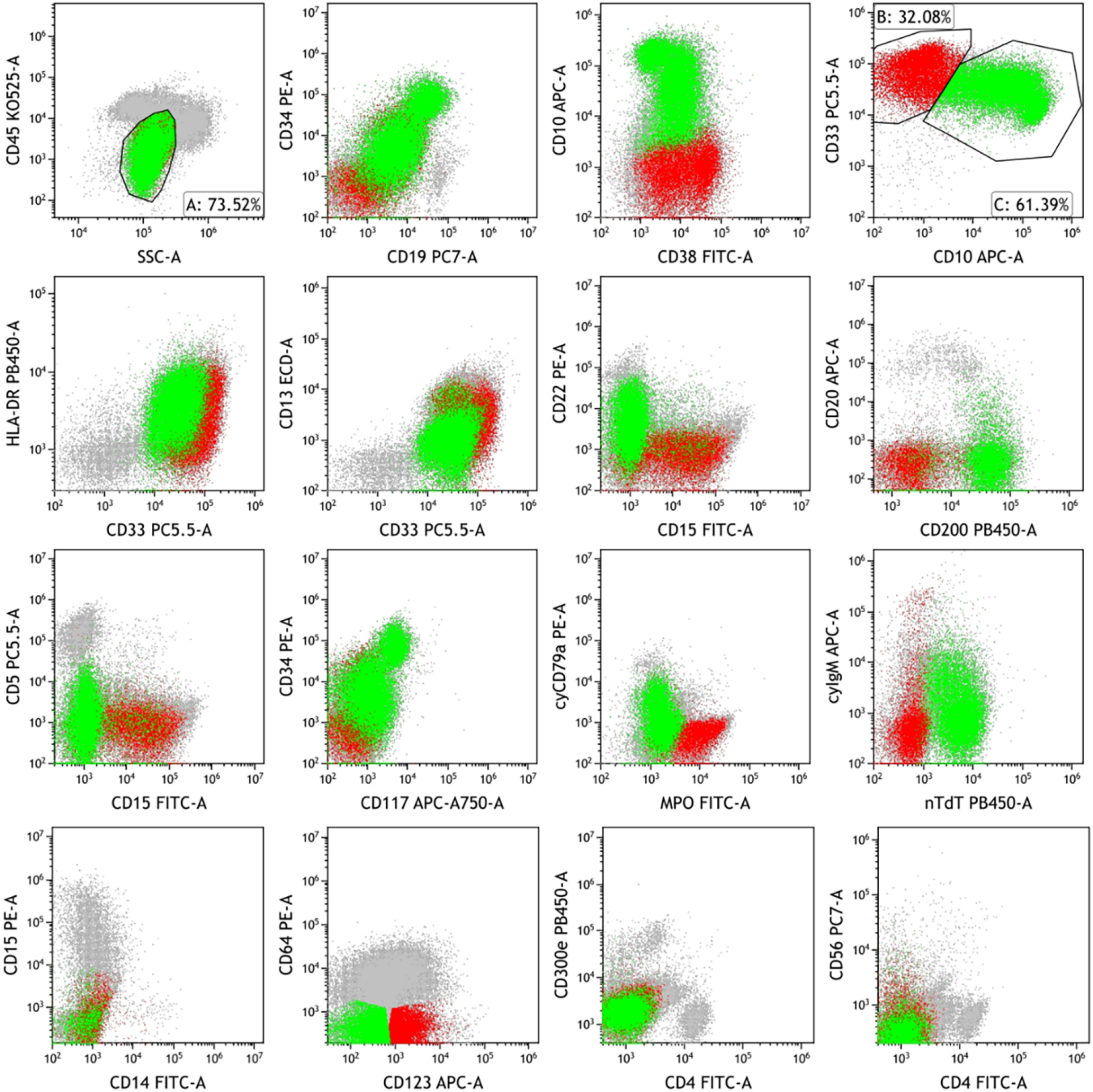
Immunophenotypic analysis by flow cytometry of bone marrow **(A)** the progenitor population. **(B)** the myeloid progenitor population. **(C)** the B-lymphoid progenitor population.

Upon admission to the hospital, she underwent all the necessary diagnostic tests. Initial laboratory evaluation demonstrated leukocytosis (total leukocyte count, 43,840/µL), anemia (hemoglobin, 9.3 g/dL), and thrombocytopenia (platelet count, 38,000/µL). The hepatitis B serological profile showed that the patient was positive for hepatitis B surface antigen, e-antibody, and core antibody. Quantitative testing for HBV DNA was negative. Quantitative polymerase chain reaction (PCR) analysis of peripheral blood detected EBV-DNA at 2.99 × 10² IU/mL. EBV-IgG was detected at a level of 642.6 U/ml. A bone marrow aspiration and biopsy were performed. Morphologic examination of the bone marrow smear revealed 28% myeloblasts and 45% immature lymphoid blasts. Immunophenotypic analysis by flow cytometry identified two distinct blast populations. The myeloid progenitor population accounted for approximately 23.59% of non-erythroid cells and expressed CD34, CD33, CD13, HLA-DR, CD38 (dim), CD123, CD15, and MPO. The B-lymphoid progenitor population accounted for approximately 45.13% of non-erythroid cells and expressed CD19 (dim), CD10, CD22, CD34, CD33, CD13, cytoplasmic CD79a, and nuclear TdT, supporting a diagnosis of MPAL (**Figure 1**). Cytogenetic analysis demonstrated the presence of the Philadelphia chromosome, t(9,22)(q34;q11.2), and fluorescence *in situ* hybridization (FISH) confirmed BCR-ABL1 rearrangement. The BCR-ABL1 transcript (p190) was detected at a level of 89%. Next-generation sequencing (NGS) did not identify additional pathogenic mutations. Bone marrow histopathology was consistent with acute leukemia. Immunohistochemistry predominantly supported B-lymphoblastic leukemia/lymphoma, with a minor component of myeloid blasts. Based on these findings, a diagnosis of B/myeloid MPAL with p190 BCR-ABL1 was established.

The patient received subsequent combination therapy with the third-generation tyrosine kinase inhibitor (TKI) olverembatinib (40 mg, every other day), the corticosteroid dexamethasone (10 mg, days 1–28), the hypomethylating agent azacitidine (100 mg, days 1–7), and the novel B-cell lymphoma 2 (Bcl-2) inhibitor lisaftoclax (200 mg on day 1, 400 mg on day 2, and 600 mg on days 3–28). After one treatment cycle, the patient achieved complete remission. Following three cycles, the BCR-ABL1 transcript (p190) became undetectable. Sustained remission has been maintained until April 2026 with regular follow-up. Given the favorable therapeutic response observed, the current regimen will be maintained with ongoing monitoring of relevant clinical indicators. Following the successful treatment, the patient expressed concern regarding the risk of relapse. She furthermore considered whether to undergo allogeneic hematopoietic stem cell transplantation or continue the current treatment. **Table 1** details the bone marrow results before and after treatment.

**Table 1:** Bone marrow results before and after treatment.

Date	Bone marrow cell morphology	Immunophenotype	Cytogenetics	BCR-ABL/ABL
Initial Diagnosis	28% myeloblasts and 45% immature lymphoid blasts	23.59% myeloid progenitor population and 45.13% B-lymphoid progenitor population	46,XX,t(9,22)(q34;q11)[8]/46,XX[2]	89%
1 month into treatment	CR	5.59% myeloid progenitor population	normal karyotype	0.0087%
2 months into treatment	CR	0.251% myeloid progenitor population	normal karyotype	0.012%
4 months into treatment	CR	negative	normal karyotype	negative

However, EBV-DNA levels, as quantified by PCR, increased over the course of three treatment cycles, reaching 5.00 × 10^4^ IU/mL, consistent with active viral replication. EBV cell-sorting analysis confirmed that the infection was localized to B cells. The patient was subsequently treated with rituximab (100 mg, administered twice), after which EBV-DNA became undetectable. After EBV-DNA became undetectable, monitoring was discontinued. **Table 2** details the EBV test before and after treatment during the case management.

**Table 2:** EBV test before and after treatment during the case management.

Date	EBV-DNA (polymerase chain reaction)	Therapy
2025.8.11 (Initial Diagnosis)	2.99×10^2^/IU/ml	–
2025.9.22	1.87×10^4^/IU/ml	–
2025.10.15	5.00×10^4^/IU/ml	rituximab 100 mg once
2025.10.27	3.73×10^2^/IU/ml	rituximab 100 mg once
2025.11.18	Below the detection limit	–

## Discussion

MPAL is a rare and diagnostically challenging entity characterized by marked biological heterogeneity. Historically, the classification systems used to identify bi-phenotypic leukemias and bi-lineal leukemias relied on the expression of lineage markers assessed by immunophenotype and cytochemistry assays, as reflected in the European Group for the Immunological Characterization of Leukemias (EGIL) classification (8). In this patient, bone marrow cell morphology test revealed 28% myeloblasts and 45% immature lymphoid blasts. Immunophenotypic analysis by flow cytometry identified two distinct blast populations. The myeloid progenitor population expressed CD33, CD13, and MPO, the B-lymphoid progenitor population expressed CD19 (dim), cytoplasmic CD79a, CD10 and CD22, supporting a diagnosis of B/myeloid bilineal leukemia. Cytogenetic analysis and molecular biology confirmed the diagnosis of B/myeloid MPAL with p190-BCR::ABL1 fusion gene. According to the expression of specific myeloid- and lymphoid-associated immunophenotypic markers, the patient met the diagnostic criteria for MPAL based on EGIL and the fifth edition of the World Health Organization classification of hematolymphoid tumors (WHO-HEM5). This case underscores the importance of bone marrow morphology, immunology, cytogenetics, molecular biology in the diagnosis of MPAL.

Compared with classical acute lymphoblastic leukemia or acute myeloid leukemia (AML), MPAL is associated with inferior outcomes, including lower remission rates, higher relapse risk, and greater therapeutic complexity. Current treatment approaches are largely extrapolated from ALL- or AML-based induction regimens, or hybrid strategies combining both. However, responses to conventional chemotherapy remain suboptimal and highly variable (9–12). Current studies revealed that patients with Ph+ MPAL should receive tyrosine kinase inhibitor-based therapy. The addition of tyrosine kinase inhibitors to the therapeutic protocol of patients with Ph+ MPAL is associated with better OS in comparison to that of other MPAL patients (3, 13). Consequently, therapy is often individualized, lacking a clear consensus on induction strategies or long-term management protocols.

In this case, the therapeutic strategy was designed to target both Ph-positive lymphoid and myeloid compartments while minimizing toxicity in the context of multiple comorbidities and chronic immunosuppression. Olverembatinib is an oral, third-generation TKI and represents the first such agent developed in China (14). Olverembatinib is an ATP-binding site inhibitor of wild-type BCR-ABL1 kinase. It demonstrates efficacy against a broad spectrum of BCR-ABL1 mutants, including T315I, which confers resistance to all first- and second-generation TKIs (15). In November 2021, olverembatinib was first approved in China for the treatment of adult patients with TKI-resistant chronic-phase CML or accelerated-phase CML who harbor the T315I mutation, confirmed by a validated diagnostic test (14). The clinical use of olverembatinib in patients with Ph+ ALL has led to an improvement in the complete remission rate, while estimated overall survival (OS) rates have also demonstrated a corresponding increase. Moreover, accumulating data suggest that the drug exhibits a favorable tolerability profile (16, 17). The recommended dosage is 40 mg administered orally every other day with meals, until disease progression or intolerable toxicity (14). As the first-in-class Bcl-2 inhibitor in China, lisaftoclax represents a novel next-generation therapeutic agent. Lisaftoclax is an orally active, potent, selective Bcl-2 inhibitor that has demonstrated significant potential for the treatment of specific hematologic malignancies (18). Notably, in July 2025, lisaftoclax received market approval in China. Despite structural differences between lisaftoclax and venetoclax, lisaftoclax exhibits stronger antitumor activity with a lower incidence of hematological toxicity (18, 19). Lisaftoclax demonstrated a favorable tolerability profile, a low incidence of infections, and no evidence of tumor lysis syndrome (19). Currently, several clinical trials are exploring the optimal dosage of lisaftoclax for patients with malignant myeloid hematological diseases, specifically investigating a dosing regimen of 200 mg on Day 1, 400 mg on Day 2, and 600 mg from Day 3 through Day 28. The combination of olverembatinib with dexamethasone, alongside lisaftoclax and azacitidine, enabled effective disease control. This case emphasizes that treatment selection in newly diagnosed MPAL requires individualized consideration of immunophenotype, cytogenetics, molecular features, patient age, performance status, and treatment tolerability.

While PTLDs are well-recognized complications of chronic immunosuppression (20), the development of *de novo* acute leukemia with concurrent EBV viremia is far less common. In transplantation recipients, EBV reactivation is typically associated with PTLD, where high EBV-DNA levels correlate with disease risk and B cells serve as the primary viral reservoir (21). In this case, the maintenance immunosuppressive regimen, consisting of tacrolimus, mycophenolate mofetil, and prednisone, facilitated the outgrowth of EBV-infected B cells. EBV-DNA levels, as quantified by PCR, increased over the course of three treatment cycles in anti-leukemic treatment, indicating that anti-leukemic therapy enhanced EBV replication. Rituximab, a monoclonal antibody drug that targets the B cell surface antigen CD20, can destroy B lymphocytes, leading to the destruction of EBV that is latent in B cells. In this case, EBV cell-sorting analysis confirmed that the infection was localized to B cells. The patient was subsequently treated with rituximab, after which EBV-DNA became undetectable. Therefore, rituximab therapy is a safe and effective approach for achieving rapid clearance of EBV DNA. It may also thereby resolve the chronic proliferation driven by persistent EBV-mediated stimulation of B lymphocytes.

This case diverges from the typical paradigm, as the primary pathology was Ph+ MPAL after kidney transplantation, yet was accompanied by high-level EBV viremia localized to B cells. This requires a unique treatment approach that should not only be effective against leukemia, but also avoid the burden on the kidneys caused by intensive chemotherapy, while controlling the infection of EBV. The successful integration of rituximab for B-cell depletion, alongside concurrent leukemia-directed therapy, highlights the importance of an individualized and pathogenesis-oriented treatment strategy. This case underscores the complexity of managing hematologic malignancies in chronically immunosuppressed patients and provides a proof-of-concept for integrated therapeutic approaches targeting both malignant and infectious processes. However, given the nature of this single-case report, these findings should be interpreted with caution. The long-term durability of response and the broader applicability of this strategy warrant further validation in larger cohorts.

## Data Availability

The original contributions presented in the study are included in the article. Further inquiries can be directed to the corresponding author.
